# Knee Osteoarthritis Therapy Targeting NLRP3 Inflammasome: Progress From Mechanism to Application

**DOI:** 10.1002/iid3.70520

**Published:** 2026-09-21

**Authors:** Wenguang Chen, Baoxiu Yi, Zhang Wang, Cong Yu, Ziru Li, Zenan Wu, Ensi Hong

**Affiliations:** ^1^ Clinical Medical College of Jiangxi University of Chinese Medicine Nanchang Jiangxi China; ^2^ The Affiliated Hospital of Jiangxi University of Chinese Medicine Nanchang Jiangxi China

**Keywords:** knee osteoarthritis, NLRP3 inflammasome, pathogenesis, therapeutic targets, traditional Chinese medicine

## Abstract

**Objective:**

This paper briefly reviews the structure, function, and activation mechanism of the NLRP3 inflammasome and its molecular role in the pathogenesis of knee osteoarthritis (KOA), and explores the potential value of traditional Chinese medicine (TCM) in treating KOA by modulating NLRP3 inflammasome activity. The review covers evidence from mechanistic studies, in vitro experiments, in vivo models, and currently available clinical‐related findings.

**Methods:**

To investigate the role of the NLRP3 inflammasome in KOA, a comprehensive literature search was conducted across multiple databases, including PubMed, MEDLINE, Web of Science, Scopus, and various Chinese biomedical repositories (CNKI, CBM, VIP, COJ). Emphasis was placed on studies published in the past 5 years concerning TCM‐based interventions targeting NLRP3 in KOA models, facilitating comparative analysis and synthesis of recent evidence.

**Results:**

NLRP3 inflammasome has been implicated in multiple pathological processes of KOA, including cartilage degradation, inflammation of the synovium, programmed cell death (pyroptosis), and the activation of osteoclasts. Interventions using TCM—such as herbal monomers, multi‐compound formulas, external preparations, and acupuncture—can mitigate these pathological events by modulating NLRP3 activity.

**Conclusion:**

The NLRP3 inflammasome is a critical mediator in KOA pathophysiology, influencing inflammation, cartilage deterioration, pyroptosis, and bone metabolism. Strategies derived from TCM that target this inflammasome may offer promising avenues for developing precise and effective treatments for KOA.

## 1 Introduction

NOD‐, LRR‐, and pyrin domain‐containing protein 3 (NLRP3) inflammasome is an intracellular complex primarily composed of pattern recognition receptors (PRRs), the adapter molecule ASC (apoptosis‐associated speck‐like protein containing a caspase activation and recruitment domain [CARD]), and the effector enzyme caspase‐1. Upon sensing pathogen‐associated molecular patterns (PAMPs) or damage‐associated molecular patterns (DAMPs), this multiprotein assembly initiates a signaling cascade that results in innate immune activation and inflammation [1]. Knee osteoarthritis (KOA), a representative degenerative joint condition, presents clinically with joint pain, stiffness in the morning, and limited mobility. Epidemiological studies have demonstrated a marked rise in KOA cases, with US prevalence having tripled since the mid‐20th century. It currently affects around 6.9% of individuals over the age of 25, amounting to over 13.7 million people [2]. In recent years, substantial progress has been made in elucidating the role of the NLRP3 inflammasome in KOA. Beyond its canonical function in host defense, aberrant activation of the NLRP3 inflammasome has increasingly been implicated in a wide range of chronic inflammatory and degenerative disorders. In particular, accumulating evidence suggests that persistent low‐grade inflammation is a critical contributor to the initiation and progression of KOA, thereby providing a mechanistic basis for linking NLRP3 inflammasome activation to joint tissue damage. Studies have demonstrated that overactivation of the NLRP3 inflammasome is closely associated with KOA progression [3, 4, 5, 6, 7], and its pathogenic effects are mainly mediated through the promotion of articular cartilage degeneration, synovial inflammation, pyroptosis, and osteoclast‐related bone destruction. In the context of KOA, “overactivation” generally refers to sustained or aberrantly enhanced activation of the NLRP3/ASC/caspase‐1 signaling axis, as evidenced by increased expression of inflammasome‐related components, elevated IL‐1β and IL‐18 release, enhanced pyroptosis, and worsening cartilage and synovial pathology. Given its central role in the pathogenesis of KOA, the NLRP3 inflammasome has emerged as a promising therapeutic target. Accordingly, interventions capable of modulating this pathway, including those derived from traditional Chinese medicine (TCM), may offer novel opportunities for the treatment of KOA. This review aims to provide a comprehensive analysis of the structural and functional aspects of the NLRP3 inflammasome, its activation pathways, and its molecular contributions to KOA pathogenesis. It further summarizes evidence from mechanistic studies, in vitro experiments, in vivo models, and currently available clinical‐related findings regarding the role and regulation of the NLRP3 inflammasome in KOA. Furthermore, it explores how TCM can modulate NLRP3 inflammasome activity, offering potential for novel therapeutic interventions. By integrating these lines of evidence, we hope to uncover targeted approaches for delaying disease progression and improving clinical outcomes in KOA.

## 2 Structure and Function of the NLRP3 Inflammasome

### 2.1 Structural Features of the NLRP3 Inflammasome

**Figure 1:**
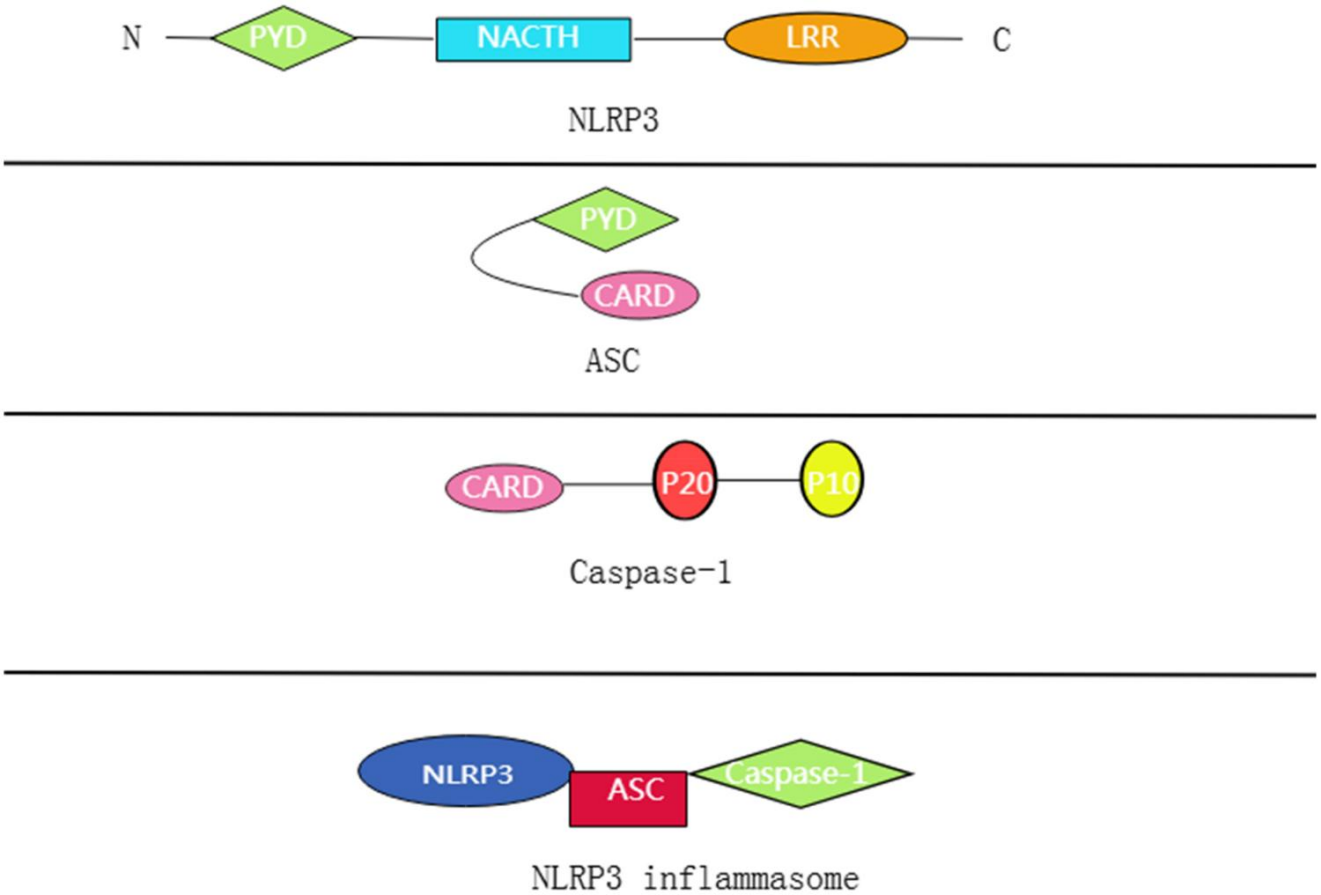
Structures of NLRP3.

The NLRP3 inflammasome is a multiprotein signaling complex composed of three essential elements: the NLRP3 sensor protein, the ASC adapter protein, and the effector enzyme caspase‐1 (Figure 1). Structurally, the NLRP3 molecule comprises three distinct domains: a pyrin domain (PYD) at the N‐terminal, a central NACHT domain (also known as NOD), and a C‐terminal leucine‐rich repeat (LRR) domain [8, 9]. Upon activation, the PYD domain of NLRP3 is exposed and interacts with the PYD domain of ASC through homotypic binding, thereby initiating downstream signaling. The LRR domain is mainly involved in molecular recognition, protein–protein interactions, and maintenance of NLRP3 autoinhibition. Conversely, the NACHT domain exhibits nucleotide (ATP/dATP) binding and hydrolysis capacities. Upon stimulation by decreased intracellular K^+^ concentrations or reactive oxygen species (ROS), the NACHT domain undergoes nucleotide‐dependent conformational rearrangements that drive NLRP3 oligomerization [10]. As an important innate immune sensor, NLRP3 detects a wide range of danger signals, including PAMPs and DAMPs, and subsequently assembles into the inflammasome complex [11]. The LRR domain is mainly involved in molecular recognition, protein–protein interactions, and maintenance of NLRP3 autoinhibition. The PYD domain forms a “PYD–PYD” linkage with ASC through homotypic interactions, whereas ASC acts as a molecular adapter by linking NLRP3 to procaspase‐1 through its PYD and CARD domains [12, 13]. Caspase‐1 itself exhibits a modular configuration, consisting of an N‐terminal CARD domain, a central large catalytic p20 subunit, and a C‐terminal small p10 subunit [14]. When the CARD domain of ASC recruits procaspase‐1 to a critical concentration, autocatalytic cleavage is triggered, ultimately generating enzymatically active caspase‐1.

The NLRP3 inflammasome consists of the sensor protein NLRP3, the adapter protein ASC, and the effector enzyme caspase‐1. NLRP3 contains an N‐terminal PYD domain, a central NACHT domain, and a C‐terminal LRR domain. ASC contains both PYD and CARD domains, whereas caspase‐1 consists of an N‐terminal CARD domain, a central p20 subunit, and a C‐terminal p10 subunit.

### 2.2 Activation Mechanism of the NLRP3 Inflammasome

**Figure 2:**
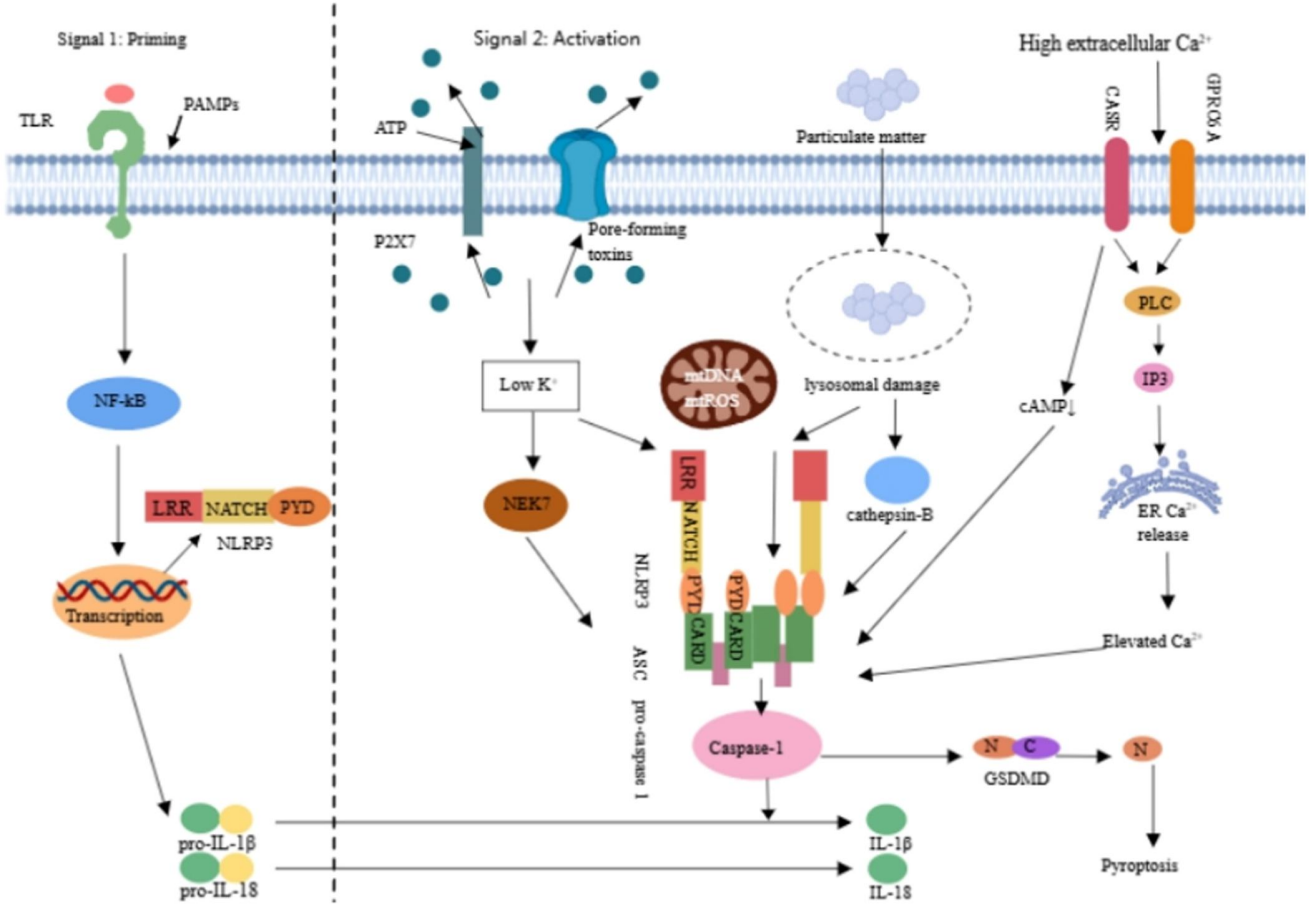
NLRP3 inflammasome activation.

The activation mechanism of NLRP3 inflammasome is relatively complex and usually requires two steps, initiation and activation (Figure 2). Initiation starts with the recognition of pathogens or danger signals by PRRs, and the interaction between PRRs and PAMPs or DAMPs leads to the activation of the nuclear factor kappa‐light‐chain‐enhancer of activated B cells (NF‐κB) pathway, which in turn regulates the elevated transcription and expression of NLRP3 and promotes the activation of the precursor NLRP3 proteins and the interleukin 1β precursor (pro‐IL‐1β), interleukin 18 precursor (pro‐IL‐18), and other precursor cytokines [15]. Recent molecular studies suggest that the priming phase is not limited to transcriptional regulation, but also involves post‐translational modifications and molecular chaperones that facilitate protein complex assembly [16, 17]. The initiation process of the NLRP3 inflammasome is highly intricate, encompassing regulation at more than just the gene transcription level, but also involves various post‐translational modification mechanisms and necessitates the involvement of specific molecular chaperones in assembling protein complexes. After completing molecular preparations for the initiation phase, the cell proceeds to the effector activation phase, activated by signals from pathogens or damage, such as extracellular adenosine triphosphate (ATP), pore‐forming toxins, viral nucleic acids, and particulate matter. Exogenous or endogenous activators induce mitochondrial dysfunction, excessive ROS generation, disruption of cellular ionic homeostasis (including K^+^ efflux and Ca^2+^ influx), and lysosomal membrane permeabilization [18, 19]. The combination of these events leads to structural changes in NLRP3 proteins, promoting their assembly into functional inflammasome complexes. ASC then recruits procaspase‐1 into the oligomerized structure, facilitating its autoproteolytic cleavage to form activated caspase‐1. This active caspase‐1 enzyme then converts the immature pro‐IL‐1β and pro‐IL‐18 into the mature cytokines IL‐1β and IL‐18. It is especially crucial to highlight that the molecular licensing mechanisms during the initiation phase, along with the stress response system in the effector phase, create a complex regulatory network that ensures precise regulation of the inflammatory response.

The NLRP3 inflammasome activation occurs through a tightly regulated two‐step mechanism: priming followed by activation. Initially, during the priming phase, PRRs, including TLR/NOD‐like receptors, detect PAMPs or DAMPs, initiating NF‐κB signaling to upregulate transcriptional expression of NLRP3 and pro‐IL‐1β precursors. Subsequently, the activation phase is triggered by danger signals such as ATP or crystalline particles, which induce critical cellular events including potassium ion (K^+^) efflux, lysosomal destabilization, and mitochondrial ROS generation. These stimuli drive NLRP3 oligomerization and recruitment of the adapter protein ASC (apoptosis‐associated speck‐like protein containing a CARD) and pro‐caspase‐1, culminating in assembly of the functional inflammasome complex capable of caspase‐1 activation and interleukin processing.

### 2.3 NLRP3 Inflammasome and Its Role in Innate Immune Responses

The core mechanism of the innate immune system, which serves as the host's initial defense against pathogens, depends on genetically encoded receptors like PRRs to initiate a swift immune response by detecting PAMPs, which are widely distributed in the plasma membrane system, endosomal structures, and cytoplasmic matrix of immune effector cells such as monocytes, macrophages, neutrophils, dendritic cells, and NK cells. These PRRs are broadly distributed across the plasma membrane system, endosomal structures, and cytoplasmic matrix of immune effector cells, including monocytes, neutrophils, dendritic cells, and NK cells. As a crucial component of the NOD‐like receptor family, the NLRP3 inflammasome is the most thoroughly investigated molecular structure [20]. The NLRP3 inflammasome is crucial for innate immunity and inflammation, acting as a key player in the intrinsic immune response and inflammatory regulation by facilitating the release of pro‐inflammatory mediators like IL‐1β and IL‐18 [21]. These cytokines can target the recruitment of immune cell populations such as macrophages and T lymphocytes, effectively reinforcing local and systemic immune defense networks. Significantly, improper activation of NLRP3 can disturb immune balance, resulting in ongoing inflammation and tissue damage. Research indicates that this state of overactivation is strongly linked to several pathological conditions, including autoimmune diseases, metabolic syndrome, and neurodegenerative disorders [22, 23, 24, 25, 26, 27, 28, 29, 30]. Thus, accurately controlling the function of NLRP3 inflammasomes is crucial for maintaining immune balance and addressing related diseases.

Taken together, the structural features, activation mechanisms, and innate immune functions of the NLRP3 inflammasome provide the biological basis for its involvement in chronic inflammatory diseases. In KOA, where cartilage degeneration, synovial inflammation, pyroptosis, and subchondral bone remodeling coexist, abnormal activation of NLRP3 may function as a molecular hub linking immune dysregulation to progressive joint destruction. Therefore, a disease‐oriented discussion of how the NLRP3 inflammasome contributes to KOA pathogenesis is warranted.

## 3 Role of NLRP3 Inflammasome in the Pathogenesis of KOA

### 3.1 NLRP3‐Mediated Degeneration of Articular Cartilage

The pathogenesis of KOA, which is characterized by degenerative changes in articular cartilage, involves multisystem interactions. Cartilage tissue degeneration involves various pathological processes, including disrupted metabolic homeostasis, breakdown of the extracellular matrix structure, chronic inflammation activation, and mechanical conduction abnormalities. Research indicates that the NLRP3 inflammasome is pathologically overexpressed in the synovial tissue of affected joints, promoting the caspase‐1‐dependent release of inflammatory mediators such as IL‐1β and IL‐18, which in turn amplifies inflammatory responses and aggravates the cycle of cartilage degradation and tissue damage [31, 32]. Under pathological conditions, increased levels of IL‐1β and tumor necrosis factor‐α (TNF‐α) greatly inhibit the production of cartilage matrix components, such as type II collagen and proteoglycans, while concurrently stimulating chondrocytes to overexpress matrix‐degrading enzymes. These encompass matrix metalloproteinase‐1 (MMP‐1), MMP‐3, MMP‐13, and a disintegrin and metalloproteinase with thrombospondin motifs (ADAMTS) [33, 34, 35]. Pathways leading to NLRP3 activation include damage to mitochondria and lysosomes, exocytosis of cytoplasmic K^+^, and oxidative stress triggered by ROS [36]. Oxidative stress causes a disruption between antioxidant enzymes and ROS scavenging systems, leading to irregular cartilage and bone metabolism, which further worsens cartilage degradation [37, 38].

From a biomechanical perspective, alterations in joint structure in KOA patients caused by abnormalities in lower limb force lines can subject local cartilage to prolonged abnormal stress. For instance, when the knee experiences inversion deformity, it leads to a heightened pressure load on the medial tibiofemoral joint, significantly disrupting the normal metabolic activity of chondrocytes due to the altered biomechanical microenvironment, thereby contributing to localized degenerative changes in cartilage. Reduced mechanical loading can successfully suppress the expression of the NLRP3 inflammasome and its downstream effectors, caspase‐1 and IL‐1β, thus slowing down cartilage degeneration, subchondral bone destruction, and secondary inflammation in the development of KOA [39]. Yang et al. [40] revealed through bioinformatics analysis that moderate mechanical stimulation can enhance cartilage health and reduce inflammation by blocking the TRAIL/NF‐κB/NLRP3 pathway, thereby improving cartilage degeneration and chondrocyte damage. Moreover, research on animal models indicates that the activation of NLRP3 disturbs the homeostasis of the cartilage matrix by enhancing the expression of proteases such as ADAMTS‐4 and MMP3/13 in tissues of ACL‐transverse osteoarthritis mice, inhibiting collagen II and aggregated proteoglycan synthesis [41].

### 3.2 NLRP3‐Mediated Synovial Inflammation

The NLRP3 inflammasome is crucial in the initiation and development of osteoarticular synovial inflammation, with immune cells frequently participating in the progression of KOA synovitis. The body's innate immune system acts as a crucial defense against pathogen invasion, with PRRs detecting and binding to DAMPs or PAMPs to create ligand polymers, which activate the innate immune response to trigger and promote osteoarthritic synovitis of the knee [42]. As a key PRR, NLRP3 interacts with DAMPs/PAMPs from synovial tissues, significantly triggering the NF‐κB signaling pathway. This cascade triggers the transcriptional release of pro‐inflammatory factors and inflammatory mediators, while also amplifying inflammation by promoting abnormal synovial cell proliferation, which ultimately exacerbates the synovial membrane pathology [43]. Zhang et al. [44] found that NLRP3 inflammasomes were present in synovial macrophages from KOA patients when stimulated by various DAMPs. The activation of the NLRP3 inflammasome specifically triggered the release of crucial inflammatory factors like IL‐1β and IL‐18 into the synovial fluid and surrounding tissues, resulting in synovial inflammation. Research indicates that MCC950, an NLRP3 inhibitor, effectively suppresses the activation of the NLRP3 inflammasome in the synovial tissue and decreases IL‐1β production [45]. Clavijo‐Cornejo et al. [46] found a 5.4‐fold increase in the expression of NLRP3 inflammasome in synovial membranes of patients with KOA, as compared to healthy controls. Sakalyte et al. [47] also observed that NLRP3 inflammasome was present in synovial fibroblasts from patients with KOA and showed high expression. The activation of the NLRP3 inflammasome enhances synovitis, playing a role in the pathological process of KOA and advancing its progression. Related studies have shown that targeted inhibition of NLRP3 inflammatory vesicle activity can effectively improve the degree of synovial inflammation and joint pain symptoms in model animals, which provides an important theoretical basis for the development of novel therapeutic strategies [48, 49, 50]. To sum up, controlling NLRP3 activity is anticipated to be an effective strategy for managing synovial inflammation and enhancing the clinical outcomes of KOA.

### 3.3 NLRP3‐Mediated Cellular Pyroptosis and Osteoclastogenesis

The NLRP3 inflammasome contributes to KOA progression by promoting chondrocyte pyroptosis and osteoclast activation. In the presence of oxidative stress, mechanical injury, or inflammatory factors, the NLRP3 inflammasome triggers a cascade that converts procaspase‐1 zymogen into its active form caspase‐1, which creates transmembrane pores by cleaving gasdermin D (GSDMD) protein, leading to the breakdown of cellular membrane integrity and the release of inflammatory mediators. Importantly, cytokines like IL‐1β and IL‐18, which are released during pyroptosis, not only attract peripheral inflammatory cells but also create a positive feedback loop that enhances the local inflammatory response. Studies have demonstrated that miRNAs can influence apoptosis, autophagy, and pyroptosis in KOA via the Wnt/β‐catenin, TGF‐β, PI3K/AKT/mTOR, and NLRP3/caspase‐1 signaling pathways [51, 52]. Following NLRP3 inflammasome activation, pro‐inflammatory cytokines such as IL‐1β and IL‐18 accelerate osteoclast differentiation and activation. This process exacerbates inflammatory responses, thereby promoting osteoclast‐mediated bone resorption activity. Concurrently, these cytokines suppress the expression of osteoblast‐associated proteins and transcription factors, contributing to impaired bone formation. Research on diabetic osteoporosis has shown that high glucose‐induced activation of the NLRP3 inflammasome in osteoclasts increases bone resorption through the MAPK/NF‐κB signaling pathway [53]. The NLRP3 inhibitor MCC950 inhibited activation of the NF‐κB signaling pathway and downregulated key factors associated with osteoclast differentiation in diabetic osteoporotic mice [54]. Curcumin blocks NLRP3 activation by inhibiting TRPM2 channel‐mediated Ca^2+^ endocytosis in an animal model, thereby attenuating chondrocyte juxtaposition [55]. Metformin reduces chondrocyte injury and cartilage degradation in a mouse model of osteoarthritis by inhibiting the activation of the NLRP3 inflammatory vesicle, which reduces cartilage degradation and slows the progression of osteoarthritis [56]. In summary, the NLRP3 inflammasome accelerates KOA progression by promoting both pyroptosis and osteoclast activation, thereby creating a vicious cycle of inflammation and tissue destruction. Targeting this pathway may therefore provide a promising therapeutic strategy for osteoarthritis. Given this broad pathogenic involvement, therapeutic strategies targeting the NLRP3 inflammasome, including TCM‐based interventions, merit further discussion (Table 1).

**Table 1:** Traditional Chinese medicine‐based interventions targeting the NLRP3 inflammasome in knee osteoarthritis.

Species	Drugs/methods	Research object	Mechanism
TCM monomer/active ingredient	Chrysin	Rats	Inhibits the activation of NLRP3 inflammasome and also reduces the levels of IL‐1β [57, 58]
	Andrographolide	Mice/chondrocytes	Regulates the circ_Rapgef1/miR‐383‐3p/NLRP3 signaling axis [59]
	Dicoumarol	Rats	Inhibits the activation of NLRP3 inflammasome [6]
	Agnuside	Rats	Inhibit the accumulation of HIF‐1α and the activation of NLRP3 inflammasome [5]
	Cardamonin	Mice	Regulating the SIRT1/p38MAPK signaling pathway reduces ROS production and NLRP3 inflammasome activation [60]
	Vanillic acid	Rats/FLS	Decreases the expression of caspase‐1, ASC, and NLRP3 and also reduce the levels of IL‐1β and IL‐18 [48]
	Casticin	Rats/FLS	Inhibits the activation of HIF‐1α/NLRP3 inflammasome [61]
	Nodakenin	Mice/chondrocytes	Regulate the mitochondrial Drp1/ROS/NLRP3 axis and inhibit the mRNA levels of COX 2, IL‐1β, and TNF‐α, as well as the expression levels of NLRP3 inflammasome and MMP13 [62]
	Crocin	SD rats	Inhibition of the stimulation of TXNIP triggers the activation of the NLRP3 inflammatory pathway [4]
	Salidroside	SD rats	Activating the Nrf2/HO‐1 antioxidant pathway inhibits the activation of NLRP3 inflammasome [63]
	Taraxasterol	SD rats	Up‐regulate the expressions of miRNA‐140 and miRNA‐146a in cartilage tissue, and inhibit the NF‐κB/TAK1 signaling pathway and the activation of the downstream NLRP3 inflammasome [64]
Traditional Chinese medicine compound	Du Huo Ji Sheng Tang	Human/rat	Inhibition of Notch1 signaling and subsequent NLRP3 activation [65]
	Yougui Yin	Rats/FLS	Promote the polarization of macrophages from M1 type to M2 type and inhibit the activation of NLRP3 inflammasome [66]
	Wufuyin	Rats	Inhibiting the activation of NLRP3 inflammasome and the pyroptosis rate of chondrocytes to improve the degeneration of articular cartilage [67]
	Huangqi Guizhi Wuwu Tang	SD rats	Reduce the expression of mRNA and protein in the inflammatory signaling pathways NLRP3 and Caspase1, and thereby inhibit the expression of inflammatory factors IL‐1β, IL‐6, and TNF‐α [68]
	Xibining	Rats	Reducing the expression of HIF‐1α and decreasing the activation of NLRP3 inflammasome [69]
	Bushen Qiangjin capsule	SD rats	Inhibit the activation of NLRP3 inflammasome in synovial tissue [70]
	Yishen Juanbi pill	SD rats	Regulating the NLRP3/NF‐κB/Caspase‐1 signaling pathway alleviates pyroptosis [71]
	Cangxitongbi capsules	SD rats	Inhibition of p38 MAPK phosphorylation reduces the expression of NLRP3, thereby leading to a decrease in the expression of its downstream Caspase‐1 and GSDMD [3]
	Chonggu granules	SD rats	Inhibiting the activation of the NLRP3/Caspase‐1/GSDMD pathway reduces the release of pro‐inflammatory factors such as IL‐1β and IL‐18 [72]
	Wutou Tang	SD rats	Down‐regulating the expression of NLRP3, Caspase‐1, IL‐1β, and IL‐18 in cartilage can thereby slow down the pyroptosis of chondrocytes [73]
	Longbie capsule	Rats	Inhibit the activation of the NF‐κB/NLRP3 pathway, thereby slowing down the pyroptosis of chondrocytes [74]
	Tongbi Jiangu recipe	SD rats	Inhibiting the phosphorylation of IκBα blocks the nuclear translocation of NF‐κB, thereby reducing the release of inflammatory mediators such as TNF‐α and COX‐2 [75]
Topical TCM formulations	“SansePowder” essential oils nanoemulsion	SD rats	Inhibits the ERS/TXNIP/NLRP3 signaling pathway and reduces the expression levels of IL‐1β and IL‐18 [76]
	Yi Ceng layer	SD rats	Improving synovial fibrosis in KOA by inhibiting the protein and gene expressions of TGF‐β1, TIMP1, and Col1A1, and suppressing the activation of NLPRP3/caspase‐1 inflammasome [77]
Acupuncture	Electroacupuncture	SD rats	Inhibits the NLRP3 inflammasome signaling pathway and reduces pyroptosis [78]
	Acupuncture and moxibustion	Rats	Regulating the NF‐κB p65/NLRP3 pathway inhibits the activation of the NLRP3 inflammasome and reduces the levels of TNF‐α, IL‐1β, and IL‐18 [79]
	Electroacupuncture	Guinea pigs	Inhibits the activation of NLRP3 inflammasome and the protein expression levels of caspase‐1 and IL‐1β [80]

## 4 Potential of NLRP3 Inflammasome as a Therapeutic Target in KOA: Insights From Traditional Chinese Medicine

Recent studies demonstrate that TCM plays a critical role in KOA treatment by targeting the NLRP3 inflammasome. TCM monomers such as vanillic acid and chrysin suppress inflammasome activation through multiple pathways, including direct NLRP3 inhibition, upstream signaling modulation, and epigenetic regulation. TCM compound prescriptions such as Du Huo Ji Sheng Tang and Yougui Yin inhibit inflammatory networks via synergistic mechanisms such as Notch1 pathway regulation and macrophage polarization modulation. Enhanced nano‐delivery systems potentiate ERS‐TXNIP pathway regulation in modified topical agents, while acupuncture mediates innate immune reprogramming through physical stimulation. These findings systematically elucidate the multitiered intervention characteristics of TCM in KOA inflammatory microenvironments, establishing a theoretical framework for future mechanistic investigations.

### 4.1 TCM Monomers/Active Ingredients

The pathological mechanism of KOA is complex and involves synovial inflammation, cartilage degradation, oxidative stress, and chondrocyte injury. Increasing evidence suggests that natural monomeric compounds derived from TCM can intervene in KOA through several convergent mechanisms centered on the NLRP3 inflammasome. Some monomers act directly on the inflammasome complex itself, whereas others primarily regulate upstream pathways related to ROS, TXNIP, HIF‐1α, or antioxidant signaling.

In the rat KOA model, vanillic acid alleviates synovitis and pain by suppressing the expression of NLRP3, ASC, and caspase‐1 and reducing IL‐1β and IL‐18 secretion [48]. Dicoumarol, in contrast, directly binds to NLRP3 and promotes its proteasomal degradation, thereby attenuating synovial inflammation and fibrosis in KOA rats [6]. Other compounds mainly interfere with upstream regulatory pathways. Cardamonin alleviates cartilage degeneration caused by iron overload by reducing ROS‐mediated NLRP3 activation, promoting SIRT1 activation, and inhibiting p38 MAPK phosphorylation [60]. Chrysin suppresses synovitis and fibrosis in rat osteoarthritic fibroblast‐like synoviocytes by inhibiting TXNIP–NLRP3 interaction through the PERK/TXNIP pathway [57, 58], whereas crocin reduces cartilage damage and improves inflammation and oxidative stress in KOA rats through TXNIP‐related inhibition of NLRP3 signaling [4]. In addition, casticin and agnuside reduce NLRP3 activation by suppressing HIF‐1α expression, thereby ameliorating synovial inflammation and fibrosis in KOA model rats [5, 61].

Because ROS is a major upstream trigger of NLRP3 activation, several monomers also exert antioxidant‐dependent protective effects. Nodakenin and CAR are involved in ROS regulation through the mitochondrial Drp1/ROS axis and the SIRT1/p38 MAPK pathway, respectively. Salidroside markedly reduces TNF‐α, IL‐1β, and MDA levels in the synovial fluid of rat KOA models by activating the Nrf2/HO‐1 antioxidant pathway; this effect is abolished in Nrf2‐knockout models, confirming Nrf2/HO‐1 as an important upstream mechanism for suppressing NLRP3 activation [63]. In addition to direct and upstream regulation, some monomers may also influence NLRP3 through epigenetic mechanisms. Andrographolide inhibits inflammasome activation through the circ_Rapgef1/miR‐383‐3p axis [59], while taraxasterol suppresses the NF‐κB/TAK1 pathway and downstream NLRP3 signaling [64]. Bicoumarin reduces the mRNA expression of fibrosis‐related markers and inflammatory cytokines, promotes NLRP3 protein degradation, and prevents inflammasome activation, suggesting complementary post‐transcriptional and post‐translational regulatory effects [62].

Although studies on individual TCM monomers are helpful for identifying specific molecular targets and signaling pathways, they may not fully capture the synergistic and holistic characteristics of TCM therapy. By contrast, compound prescriptions may exert broader regulatory effects through coordinated actions on multiple targets and biological processes.

### 4.2 Traditional Chinese Medicine Compound

In recent years, TCM formulas have shown unique potential in KOA therapy through multitarget and multilevel regulation. Although individual formulas differ in composition and emphasis, many share common protective effects, including suppression of NLRP3 inflammasome‐related inflammatory signaling, attenuation of pyroptosis, reduction of IL‐1β and IL‐18 release, and preservation of cartilage integrity. On this basis, different formulas may exert additional therapeutic effects through distinct upstream pathways or microenvironmental regulation.

A major pattern among TCM formulas is the inhibition of NLRP3‐related pyroptotic signaling. Chonggu granules decrease the release of pro‐inflammatory factors by blocking the NLRP3/caspase‐1/GSDMD axis, thereby enhancing cell survival and reducing matrix degradation [72]. Similarly, Cangxitongbi capsules lower NLRP3 expression by inhibiting p38 MAPK phosphorylation, which reduces downstream caspase‐1 and GSDMD expression and slows chondrocyte pyroptosis in KOA model rats [3]. Yishen Juanbi pill also alleviates cartilage injury by modulating the NLRP3/NF‐κB/caspase‐1 pathway [71], whereas Wutou Tang reduces the levels of NLRP3, caspase‐1, IL‐1β, and IL‐18 in cartilage, thereby suppressing chondrocyte pyroptosis and delaying cartilage degeneration [73].

Beyond these shared antipyroptotic effects, several formulas act through distinct upstream mechanisms. Du Huo Ji Sheng Tang reduces cartilage breakdown in KOA mice by blocking Notch1 signaling, an upstream regulator of NLRP3 activation [65]. Longbei capsules suppress the NF‐κB/NLRP3 pathway by targeting MYD88 through miR‐203a‐3p, thereby reducing cartilage extracellular matrix degradation and chondrocyte injury [74]. In addition, Tongbi Jiangu recipe suppresses inflammation and delays cartilage degeneration by inhibiting IκBα phosphorylation and reducing TNF‐α and COX‐2 release [75], whereas Wufuyin mainly acts through ROS clearance to attenuate inflammasome activation and chondrocyte pyroptosis [67]. Huangqi Guizhi Wuwu Tang also decreases NLRP3‐ and caspase‐1‐related inflammatory signaling and reduces IL‐1β, IL‐6, and TNF‐α expression, with the high‐dose group showing efficacy comparable to meloxicam [68].

Recent studies have further highlighted the role of TCM formulas in regulating the immune microenvironment of KOA. Yougui Yin improves the cartilage microenvironment by promoting macrophage polarization from the pro‐inflammatory M1 phenotype to the anti‐inflammatory M2 phenotype, lowering IL‐1β and IL‐6 levels in the joint cavity and inhibiting chondrocyte apoptosis and pyroptosis [66]. Xibining alleviates synovial inflammation by counteracting hypoxia in the synovial microenvironment, reducing HIF‐1α expression, and suppressing NLRP3 activation [69]. Bushen Qiangjin capsule reduces synovial inflammation and fibrosis in KOA rats by inhibiting NLRP3 inflammasome activation in synovial tissues, thereby attenuating inflammasome‐driven pyroptosis and the inflammatory cascade [70].

In addition to orally administered herbal formulas, topical TCM formulations represent another important therapeutic approach for KOA. By acting more directly on the local joint microenvironment, these preparations may provide complementary advantages in regulating NLRP3‐associated inflammatory responses.

### 4.3 Topical TCM Formulations

Topical TCM formulations ameliorate synovial inflammation in KOA mainly by suppressing local NLRP3 inflammasome‐related inflammatory signaling. Zhang et al. [77] reported that this type of topical therapy reduced the levels of IL‐1β, TNF‐α, MMP‐1, and MMP‐13 and showed anti‐inflammatory effects comparable to those of caspase‐1 inhibitors, indicating both anti‐inflammatory and cartilage‐protective properties. In addition, Liu et al. [76] developed NEs‐SP‐EO by integrating the volatile oil of traditional Sanse powder with nanotechnology and demonstrated that it acts through the ERS/TXNIP–NLRP3 axis to reduce IL‐1β and IL‐18 release, while also improving transdermal delivery efficiency and targeting. Together, these findings suggest that external TCM therapies share a common anti‐inflammatory basis, whereas formulation innovation may provide additional advantages in local drug delivery and pathway‐specific intervention.

Different from topical herbal preparations, acupuncture exerts its effects mainly through physical stimulation and neuroimmune modulation. This distinctive mode of action offers another complementary strategy by which TCM may influence NLRP3 inflammasome activity in KOA.

### 4.4 Acupuncture

The mechanisms underlying acupuncture as a nonpharmacological treatment for KOA have attracted increasing attention in recent years. Available studies suggest that acupuncture may delay KOA progression by modulating NLRP3 inflammasome‐related inflammatory signaling and pyroptosis‐associated pathways [78, 79, 80]. Among different acupuncture‐based interventions, electroacupuncture has been shown to reduce mechanically abnormal pain through inhibition of the NLRP3/caspase‐1/IL‐1β axis [80], while also delaying cartilage degradation by decreasing the expression of the pyroptosis‐related protein GSDMD [78]. In addition, moxibustion appears to exert anti‐inflammatory effects through coordinated regulation of the NF‐κB/NLRP3 pathway, accompanied by changes in ASC and serum IL‐18 levels [79]. Although most evidence is currently derived from animal studies, the available data consistently suggest that acupuncture may protect joint tissues by regulating the innate immune‐inflammatory response network.

## 5 Conclusions and Perspectives

To sum up, the NLRP3 inflammasome influences the onset, progression, and resolution of KOA by regulating cartilage matrix degradation, synovial inflammation, pyroptosis, and the imbalance between bone resorption and bone formation, thereby contributing to a pathological cycle of matrix destruction, inflammatory amplification, and disordered bone metabolism. In recent years, TCM has shown distinct advantages in KOA treatment by targeting the NLRP3‐related inflammatory signaling. Across different intervention forms—including monomers, formulas, topical preparations, and acupuncture—TCM appears to exert convergent anti‐inflammatory and joint‐protective effects, while individual therapies may additionally target specific upstream regulators, immune microenvironmental changes, or delivery efficiency.

However, several limitations remain. First, the activation mechanism of NLRP3 has not been fully elucidated, especially the causal relationship between metabolic abnormalities, such as mitochondrial dysfunction, and inflammasome activation. Second, most studies on TCM are still confined to animal models, and the relationship between complex components and their molecular targets requires further clarification through more rigorous approaches, including component knockout strategies and molecular docking. In addition, the interactions between NLRP3 and other inflammatory pathways, such as NF‐κB and MAPK, as well as its dynamic changes during different stages of KOA, have not yet been systematically characterized.

Future studies may focus on three directions. First, single‐cell sequencing and organoid models could be used to define the cell‐specific regulatory network of NLRP3 in chondrocytes, synoviocytes, and other joint‐related cell populations. Second, nanodelivery systems targeting NLRP3 may enhance the local accumulation and therapeutic efficiency of TCM‐derived agents in the joint. Third, translational clinical studies are needed to establish an integrated evaluation system for NLRP3 activation and therapeutic efficacy by combining imaging‐based methods with biomarkers. Through multidisciplinary integration, these advances may contribute to more precise classification and treatment of KOA and offer new strategies for delaying joint degeneration.

## Author Contributions

**Wenguang Chen:** conceptualization, investigation, writing – original draft, and visualization. **Baoxiu Yi:** investigation, writing – original draft. **Zhang Wang:** investigation, writing – review and editing. **Cong Yu:** writing – review and editing. **Ziru Li:** formal analysis, investigation. **Zenan Wu:** investigation. **Ensi Hong:** writing – review and editing, project administration, conceptualization, supervision, funding acquisition.

## Conflicts of Interest

The authors declare no conflicts of interest.

## Data Availability

This article is a review and does not report original data. Therefore, no new data were generated or analyzed in this study.
